# Neoadjuvant TRK inhibitors versus chemotherapy in advanced NTRK fusion-positive sarcomas: a real-world evidence analysis

**DOI:** 10.1093/oncolo/oyaf401

**Published:** 2025-12-05

**Authors:** Fuyi Zhu, Haiyan Cheng, Yali Han, Shen Yang, Zhiyun Zhu, Qinghua Ren, Yan Su, Zhenni Wang, Hong Qin, Wei Yang, Shan Wang, Yijin Gao, Huanmin Wang

**Affiliations:** Department of Surgical Oncology, Key Laboratory of Major Diseases in Children, Ministry of Education, Beijing Children’s Hospital, Capital Medical University, National Center for Children’s Health, Beijing 100045, China; Department of Surgical Oncology, Key Laboratory of Major Diseases in Children, Ministry of Education, Beijing Children’s Hospital, Capital Medical University, National Center for Children’s Health, Beijing 100045, China; Department of Oncology, Shanghai Children’s Medical Center, Shanghai Jiao Tong University School of Medicine, Shanghai 200127, China; Department of Surgical Oncology, Key Laboratory of Major Diseases in Children, Ministry of Education, Beijing Children’s Hospital, Capital Medical University, National Center for Children’s Health, Beijing 100045, China; Department of Surgical Oncology, Key Laboratory of Major Diseases in Children, Ministry of Education, Beijing Children’s Hospital, Capital Medical University, National Center for Children’s Health, Beijing 100045, China; Department of Surgical Oncology, Key Laboratory of Major Diseases in Children, Ministry of Education, Beijing Children’s Hospital, Capital Medical University, National Center for Children’s Health, Beijing 100045, China; Department of Medical Oncology, Pediatric Oncology Center, National Center for Children’s Health, Key Laboratory of Pediatric Hematology Oncology, Key Laboratory of Major Diseases in Children, Ministry of Education, Beijing Children’s Hospital, Capital Medical University, Beijing 100045, China; Department of Pediatric Surgical Oncology, Children’s Hospital of Chongqing Medical University, Chongqing 400014, China; Department of Surgical Oncology, Key Laboratory of Major Diseases in Children, Ministry of Education, Beijing Children’s Hospital, Capital Medical University, National Center for Children’s Health, Beijing 100045, China; Department of Surgical Oncology, Key Laboratory of Major Diseases in Children, Ministry of Education, Beijing Children’s Hospital, Capital Medical University, National Center for Children’s Health, Beijing 100045, China; Department of Pediatric Surgical Oncology, Children’s Hospital of Chongqing Medical University, Chongqing 400014, China; Department of Oncology, Shanghai Children’s Medical Center, Shanghai Jiao Tong University School of Medicine, Shanghai 200127, China; Department of Surgical Oncology, Key Laboratory of Major Diseases in Children, Ministry of Education, Beijing Children’s Hospital, Capital Medical University, National Center for Children’s Health, Beijing 100045, China

**Keywords:** real-world multicenter analysis, chemotherapy, TRK inhibitors, NTRK fusion-positive sarcomas, treatment failure

## Abstract

**Importance:**

Despite the proven efficacy of tropomyosin receptor kinase (TRK) inhibitors in advanced neurotrophic tyrosine receptor kinase (NTRK) fusion-positive sarcomas, chemotherapy remains the default first-line therapy in many developing countries.

**Objective:**

This real-world study directly compares outcomes of TRK inhibitors with chemotherapy.

**Design:**

This was a multicenter, retrospective cohort study.

**Setting:**

50 children with advanced NTRK fusion-positive sarcomas were analyzed from three study centers (2018-2024).

**Intervention:**

Patients were assigned into two groups according to their choice of treatment,including chemotherapy or TRK inhibitors..

**Main outcomes and Measures:**

Endpoints included treatment failure rate, objective response rate (ORR), mutilating surgery, time to treatment failure, and event-free survival (EFS). Subgroup analyses were conducted for infantile fibrosarcoma (IFS) and NTRK-rearranged spindle cell tumors.

**Results:**

The efficacy of TRK inhibitors (*n* = 37) was markedly superior compared to chemotherapy (*n* = 13). Treatment failure was almost eliminated (2.7% vs. 61.5%, *P* < 0.001), and ORR was significantly higher (91.9% vs. 53.8%, *P* = 0.006). Subgroup analysis revealed that TRK inhibitors prevented mutilating surgery in IFS (0.0% vs. 42.9%, *P* < 0.001) and improved the ORR in NTRK-rearranged spindle-cell tumors (95.0% vs. 0.0%, *P* < 0.001) in which chemotherapy was ineffective. TRK inhibition also induced faster tumor shrinkage, smaller preoperative burden, and 40% pathological complete responses. Finally, TRK inhibitor also prolonged the time-to-treatment failure and EFS.

**Conclusions and Relevance:**

Upfront TRK inhibition provided faster, deeper responses, avoided mutilating surgeries, and enabled curative resections. These findings support TRK inhibitors as the preferred first-line option for children with NTRK fusion-positive sarcomas.

Implications for Practice In children with NTRK fusion-positive sarcomas, upfront TRK inhibition yielded faster, deeper responses and eliminated mutilating surgery in our cohort. These data support TRK inhibitors as preferred neoadjuvant options, particularly to facilitate non–morbid resections and to avoid functional disability.

## Introduction

NTRK fusion-positive sarcomas are a class of malignancies characterized by gene rearrangements involving the neurotrophic tyrosine receptor kinase (NTRK) family (NTRK1, NTRK2, and NTRK3), which represent a key mechanism of oncogenic activation.^1^ These fusions occur at high frequency in certain pediatric malignancies. Infantile fibrosarcoma (IFS) is the prototypical example, with >90% of cases harboring NTRK fusions—most commonly ETV6-NTRK3—whereas the recently recognized NTRK-rearranged spindle-cell tumors represent another clinically important subtype within the same molecular category.^2–4^ Localized NTRK fusion-positive sarcomas are usually curable with surgery surgically, but once tumors infiltrate vital structures or metastasize, the risk of surgical morbidity and local recurrence increases, making effective systemic therapy indispensable.

European pediatric STS Study Group (EpSSG) used vincristine and actinomycin-D (VA) as neoadjuvant chemotherapy in IRS III IFS patients . The objective response rate (ORR) was 68%. Three of twenty-five patients underwent mutilating surgery.^5^ Three-year event-free survival (EFS) was 84%. Likewise, the Cooperative Weichteilsarkom Studiengruppe (CWS) reported that VA and cyclophosphamide (VAC) regimen achieved an ORR of 75% (21 of 28 IRS III patients), with a 5-year EFS of 81%,^6^ however, still 3 cases received amputation. Thus, neoadjuvant chemotherapy is effective for most advanced disease, but 20%-30% derive little benefit, while short- and long-term side-effects should not be ignored. Furthermore, we found that NTRK-rearranged spindle-cell tumors are typically insensitive to conventional chemotherapy, highlighting the limitations of current cytotoxic strategies and underscoring the need for safer, more effective, molecularly targeted approaches.

Encouragingly, highly selective TRK inhibitors have transformed the therapeutic landscape for NTRK fusion-positive sarcomas. Larotrectinib produces pronounced and durable tumor responses^7^^,^^8^ with 90.2% ORR in advanced IFS^9^ and 71% in NTRK-rearranged spindle-cell tumors, respectively.^10^ Additionally, Entrectinib is a multi−targeted, pan-TRK and ROS1 inhibitor that, in a pooled analysis of three pediatric studies, has an ORR of 91.7% among 24 patients with NTRK fusion-positive extracranial solid tumors.^11^ However, nearly all TRK-inhibitor data derive from multi−institutional, single-arm basket trials without a concurrent chemotherapy group. Direct efficacy comparisons between vincristine-based chemotherapy and targeted therapy are challenging in NTRK fusion-positive sarcomas.

In this study, we conducted a multicenter real-world study across three Chinese pediatric oncology centers to compare (1) the treatment failure rate (disease progression or mutilating surgery), (2) ORR, (3) the rate of treatment-related mutilation, and (4) subgroup efficacy in IFS versus spindle-cell tumors between neoadjuvant chemotherapy and neoadjuvant TRK inhibitor therapy. We sought to explore the optimal preoperative strategy for children with NTRK fusion-positive sarcomas.

## Methods

### Study design and setting

This retrospective, multicenter, real-world study included 50 pediatric patients with NTRK fusion-positive sarcomas from three tertiary pediatric hospitals in China between January 2018 and January 2024. The distribution of cases was as follows: Beijing: 46 cases; Shanghai: 3 cases; Chongqing: 1 case. Patients were followed through January 1, 2025. This multicenter study was approved by the institutional review boards of Beijing Children’s Hospital, Shanghai Children’s Medical Center and Children’s Hospital of Chongqing Medical University, and was conducted in accordance with the Declaration of Helsinki. Written informed consent was obtained from the parents or legal guardians of all participants. The study was done according to the Strengthening the Reporting of Observational Studies in Epidemiology (STROBE) statement guidelines for observational studies (Supplementary Table S1).

### Eligibility and exclusion criteria

All included children were younger than 18 years at diagnosis. All tumors were pathologically confirmed NTRK fusion- positive by FISH, RT-PCR, or NGS. Only cases classified as locally advanced, unresectable (IRS III/IV), or metastatic were eligible for this study.

Patients who had received prior systemic anti-tumor therapy, including chemotherapy, targeted therapy, or radiotherapy, were excluded, however, patients who had previously undergone definitive surgery alone and subsequently experienced local relapse were eligible for inclusion. All patients received neoadjuvant chemotherapy or neoadjuvant TRK inhibitor (Larotrectinib or Entrectinib or Zurletrectinib) before definitive surgery. Complete baseline and post−treatment imaging were required. Patients with concomitant malignancy or follow-up duration of less than 3 months after treatment initiation were also excluded.

### Treatment groups

Chemotherapy cohort: we used alternating VAC/IE regime for at least 4 cycles before surgery. VAC: Vincristine (1.5 mg/m^2^, day 1, day 8, and day 15), Actinomycin-D (1.5 mg/m^2^, maximally administered dose 2.5 mg, day 1), Cyclophosphamide (1.2 g/m^2^, day 1). IE: Ifosfamide (1.8 g/m^2^, days 1-5), Etoposide (100 mg/m^2^, days 1-5).

One patient with osteosarcoma received a standard osteosarcoma-specific chemotherapy regimen (MAP), consisting of Methotrexate, Adriamycin and Cisplatin, in accordance with established osteosarcoma treatment protocols. Targeted cohort: Larotrectinib (100 mg/m^2^, bid) or Entrectinib (300 mg/m^2^, qd) or Zurletrectinib (7.2 mg/m^2^, qd) until maximal response or surgery. One cycle of therapy was 28 days.

### Timing of surgery

The decision to proceed with surgery was made through multidisciplinary team (MDT) discussion, taking into account tumor response, resectability, functional risk, and the parental preference. Surgery was performed only for the primary lesion, and metastatic sites were not included in the surgical plan. If parents requested surgery, surgery was deferred until maximal tumor response or disease stabilization was achieved and was subsequently approved by the MDT to ensure safety and feasibility.

### Data collection

A total of 8 baseline demographic and clinicopathologic variables were abstracted from the electronic medical records, including age at diagnosis, primary tumor site, maximal tumor diameter, initial or relapsed status, presence of distant metastasis, histological subtype (IFS, NTRK-rearranged spindle-cell tumor, other), surgery performed, and treatment completion status.

### Endpoints and definitions

The primary endpoint was treatment failure rate, defined as either radiographic or clinical disease progression or the requirement for mutilating surgery (procedures causing major functional impairment or disfigurement, such as limb amputation, nerve sacrifice, or facial deformity). The secondary efficacy endpoints were ORR (the rate of complete remission plus partial remission) and disease-progression rate (DPR). Remission, defined per RECIST v1.1 as PR or CR, or as radiographic no evidence of disease (NED), was limited to responses achieved with systemic therapy alone and did not include remission following surgery. Time to first response was defined as the time interval from treatment initiation to the first documented remission. DPR was defined as the proportion of patients experiencing disease progression during the neoadjuvant treatment period, based on RECIST v1.1 criterion. Furthermore, EFS was defined as the time from diagnosis to disease progression, death, relapse or last follow-up. For analysis of time-to-treatment failure, an event was defined as disease progression or mutilating surgery. Radiographic response was independently evaluated by two blinded radiation oncologists in accordance with RECIST v1.1, using paired pre− and post−treatment imaging studies for comparison.

### Statistical analysis

The statistical analysis was performed using SPSS, version 24 (IBM). Data were first analyzed for normality using the Shapiro−Wilk normality test. Continuous variables with a normal distribution were presented as mean ± standard deviation and compared between groups using independent-samples Student’s *t*-test. Non–normally distributed data were expressed as median (range) and Mann−Whitney U or Kruskal−Wallis tests were used for group comparisons. Categorical data were expressed as numbers and percentages, which compared using Fisher’s exact. Time-to-treatment failure and EFS were compared using log-rank tests. Differences were considered statistically significant when *P*-values were ≤0.05.

## Results

### Baseline characteristics were comparable between treatment groups

A total of 50 patients were included in this study. The TRK inhibitor cohort comprised 37 patients, including 22 who received larotrectinib, 14 who received entrectinib, and 1 who received zurletrectinib.The median follow-up time was 22.9 months (95% CI: 20.1-25.8). The chemotherapy group (*n* = 13) received an alternating VAC/IE regime had a median follow-up time of 59.4 months. There were no significant differences in age or maximal tumor diameter between the two groups. The most common histologic subtypesin the chemotherapy and TRK inhibitor groups were IFS (7/13, 53.8%) and NTRK-rearranged spindle-cell tumor (20/37, 54.1%), respectively. The limbs were the most common primary tumor site in both groups. In addition, 15.4% (2/13) of patients in the chemotherapy group had distant metastasis, including lung metastasis, whereas all patients in the TRK inhibitor group had locally advanced disease. It was worth noting that except one patient in the chemotherapy group who was lost to follow-up after tumor progression, 12/13 (92.3%) completed therapy. Of these, 91.7% (11/12) underwent definitive surgery for tumor removal, and one patient achieved long-term tumor-bearing survival without surgery. In contrast, 56.8% (21/37) of patients in the TRK inhibitor group remained on treatment at the end of follow-up, and only 10 patients received surgical resection. A full comparison of baseline characteristics between the 2 groups is listed in Table 1.

**Table 1.** The baseline characteristics of the targeted and chemotherapy group were compared.

Characteristics	Patients (*n* = 50)	Chemotherapy (*n* = 13)	Targeted therapy (*n* = 37)	*P*-value
**Age, months**				
**Median (IQR)**	10.5 (2-48)	12 (5-48)	9 (2-45)	0.791
**Maximal tumor diameter, cm**				
**Mean ± SD**	6.47 ± 2.95	6.62 ± 2.85	6.42 ± 3.02	0.300
**Initial or relapsed, *n* (%)**				0.531
** Initial**	33 (66.0)	10 (76.9)	23 (62.2)	
** Relapsed**	17 (34.0)	3 (23.1)	14 (37.8)	
**Histology, *n* (%)**				0.292
** IFS**	21 (42.0)	7 (53.8)	14 (37.8)	
** NTRK-rearranged spindle-cell tumors**	25 (50.0)	5 (38.5)	20 (54.1)	
** Osteosarcoma**	1 (2.0)	1 (7.7)	0 (0.0)	
** Spinal cord tumors**	2 (4.0)	0 (0.0)	2 (5.4)	
**Hemispheric infantile glioma**	1 (2.0)	0 (0.0)	1 (2.7)	
** Site, *n* (%)**				0.072
** Head and face**	6 (12.0)	0 (0.0)	6 (16.2)	
** Limbs**	27 (54.0)	9 (69.2)	18 (48.6)	
** Trunk**	9 (18.0)	1 (7.7)	8 (21.6)	
** Chest cavity**	1 (2.0)	0 (0.0)	1 (6.5)	
** Abdominal cavity**	2 (4.0)	2 (15.4)	0 (0.0)	
** Pelvic cavity**	2 (4.0)	1 (7.7)	1 (0.0)	
** CNS**	3 (6.0)	0 (0.0)	3 (8.1)	
**Distant metastasis, *n* (%)**				0.107
** Locally advanced**	48 (96.0)	11 (84.6)	37 (100%)	
** Metastatic**	2 (4.0)	2 (15.4)	0 (0.0)	
**Treatment disposition, *n* (%)**				0.003
** Ongoing**	29 (58.0)	1 (7.7)	21 (56.8)	
** Discontinued**	21 (42.0)	12 (92.3)	16 (43.2)	
**Surgery, n (%)**	*n* = 49	*n* = 12	*n* = 37	<0.001
** Yes**	21 (42.9)	11 (91.7)	10 (27.0)	
** No**	28 (57.1)	1 (8.3)	27 (73.0)	

Abbreviation: IQR, interquartile range.

### TRK inhibitors significantly improved the neoadjuvant treatment efficacy

A total of 9 treatment failure events were observed among 50 patients, including 6 cases of tumor progression (5 in the chemotherapy group and 1 in the targeted group) and 3 cases requiring mutilating surgery, all in the chemotherapy group, characterized by postoperative limping. The treatment failure rate in the chemotherapy group was significantly higher than that in the targeted group (61.5% [8/13] [95% CI: 30.9%-92.1%] vs. 2.7% [1/37] [95% CI: −2.8% to 8.2%]; *P* < 0.001). In terms of best overall response, CR occurred exclusively in the targeted group (9/37, 24.3%) and were not seen with chemotherapy (0/13). ORR of the latter was 53.8% (7/13) [95% CI: 22.5%-85.2%], while that in the former was 91.9% (34/37) [95% CI: 82.7%-101.1%]. Median time to first radiological response did not differ significantly between groups (72.4 ± 41.1 days for targeted therapy vs. 87.4 ± 34.7 days for chemotherapy; *P* = 0.371). In addition, no patient in the targeted group required mutilating surgery, whereas 23.1% (3/13) [95% CI: −3.4% to 49.6%] of chemotherapy patients did (*P* = 0.015). Detailed results of efficacy comparisons were shown in Table 2.

**Table 2.** Comparison of treatment efficacy between the TRK inhibitor and chemotherapy groups.

	Patients (*n* = 50)	Chemotherapy (*n* = 13)	Targeted therapy (*n* = 37)	*P*-value
**Response, *n* (%)**				0.003
** CR**	9 (18.0)	0 (0.0)	9 (24.3)	
** PR**	32 (64.0)	7 (53.8)	25 (67.6)	
** SD**	3 (6.0)	1 (7.7)	2 (5.4)	
** PD**	6 (12.0)	5 (38.5)	1 (2.7)	
**Days to first response** ± **SD**	74.90 ± 40.06	87.43 ± 34.66	72.4 ± 41.05	0.371
**Reason for failed, *n* (%)**	*n* = 9	*n* = 8	*n* = 1	
** Tumor progression**	6 (66.7)	5 (62.5)	1 (100)	
** Mutilating surgery**	3 (33.3)	3 (37.5)	0 (0.0)	
**Objective response rate**	82.0% (95% CI: 71.0-93.0)	53.8% (95% CI: 22.5-85.2)	91.9% (95% CI: 82.7-101.1)	0.006
**Treatment failure rate**	18.0% (95% CI: 7.0-29.0)	61.5% (95% CI: 30.9-92.1)	2.7% (95% CI: −2.8 to 8.2)	<0.001
**Disease progression rate**	12.0% (95% CI: 2.7-21.3)	38.5% (95% CI: 14.8-77.5)	2.7% (95% CI: −2.8 to 8.2)	0.003
**Mutilating surgery rate**	8.0% (95% CI: 0.2-15.8)	23.1% (95% CI: −3.4 to 49.6)	0.00%	0.015

### Subgroups analyses of clinical effective rate

IFS and NTRK-rearranged spindle-cell tumors are the two main pathological types of NTRK fusion-positive sarcomas, and their responses to standard chemotherapy differ markedly. In the IFS subgroup (median age, 6.2 months), both chemotherapy and TRK inhibitors achieved excellent tumor control. The ORR was 92.9% (13/14, 95% CI: 77.4%-108.3%) with TRK inhibitors versus 100% (7/7) with chemotherapy. However, chemotherapy was associated with a 42.9% (3/7, [95% CI: 7.7-106.6]) incidence of mutilating surgery, whereas no such events occurred after targeted therapy (*P* < 0.001). TRK inhibitor therapy also reduced the number of treatment cycles needed to reach remission (median, 2 vs 4; *P* = 0.012). In NTRK-rearranged spindle-cell tumors, outcomes diverged sharply. Chemotherapy yielded no objective responses and an 80% (4/5, 95% CI: 24.5%-135.5%) disease progression rate, whereas TRK inhibitors produced a 95.0% (19/20, 95% CI: 84.5-105.5) ORR and no progression events were observed in this subgroup (both *P* < 0.001). A full subgroup analyses between these 2 groups is listed in Table 3.

**Table 3.** Comparative efficacy of chemotherapy and TRK inhibitors in IFS and NTRK-rearranged spindle-cell tumors.

Subgroup analysis	Patients (*n* = 46)	Chemotherapy (*n* = 12)	Target therapy (*n* = 34)	*P*-value
**Disease progression, *n* (%)**				
** IFS**	1/21 4.8% (95% CI: −5.2 to 14.7)	0/7 0.0%	1/14 7.1% (95% CI: −8.3 to 22.6)	1.000
**NTRK-rearranged spindle-cell tumors**	4/25 16.0% (95% CI: 0.6-31.4)	4/5 80% (95% CI: 24.5-135.5)	0/20 0.0%	<0.001
**Mutilating surgery, *n* (%)**				
** IFS**	3/21 14.3% (95% CI: −0.2 to 30.6)	3/7 42.9% (95% CI: 7.7-106.6)	0/14 0.0%	0.026
**NTRK-rearranged spindle-cell tumors**	0/25 0.0% (95% CI: −4.5 to 12.8)	0/5 0.0%	0/20 0.0%	1.000
**Objective response rate, *n* (%)**				
** IFS**	20/21 95.2% (95% CI: 85.3-105.2)	7/7 100%	13/14 92.9% (95% CI: 77.4-108.3)	1.000
**NTRK-rearranged spindle-cell tumors**	19/25 76.0% (95% CI: 58.0-94.0)	0/5 0.0%	19/20 95.0% (95% CI: 84.5-105.5)	<0.001
**Remission cycle, median (IQR)**				
** IFS**	2 (2-4)	4 (3-6)	2 (2-3)	0.012
**Days to remission, median (IQR)**				
** IFS**	63 (55-97)	84 (60-126)	60 (54-90)	0.197

Abbreviations: IQR, interquartile range.

### Neoadjuvant TRK inhibitors led to faster and deeper responses

After excluding 2 patients who switched to targeted therapy following disease progression on chemotherapy and subsequently underwent surgery, 9 chemotherapy-treated and 10 targeted therapy-treated patients who received definitive surgery were included for further analysis. The 2 groups were comparable in terms of age, maximal tumor diameter, tumor site, and presence of distant metastasis. The median time from diagnosis to remission was nearly 1 month shorter in the targeted group (50.4 ± 33.14 days vs. 84.5 ± 37.01 days), accompanied by significant tumor shrinkage prior to surgery (median size: 1.4 ± 0.96 cm vs. 4.61 ± 3.16 cm, *P* = 0.007). Moreover, the targeted group achieved a higher proportion of R0 resections (80% vs. 66.7%; *P* = 0.777) and showed pathological complete response (pCR) in 4 of 10 tumors (40%), whereas no pCRs were observed in the chemotherapy group (*P* = 0.087). Furthermore, mutilating surgery was accepted in 3 of 9 chemotherapy patients (33%) but in none of those receiving TRK inhibitors (*P* = 0.087). A full comparison of preoperative tumor response, resection quality, and pathological remission between the 2 groups is summarized in Table 4.

**Table 4.** Preoperative tumor response and surgical outcomes following neoadjuvant TRK inhibitors versus chemotherapy.

Characteristics	Patients (*n* = 19)	Chemotherapy (*n* = 9)	Target therapy (*n* = 10)	*P*-value
**Age, months**				
**Median (IQR)**	7.0 (1.5-31.0)	8 (1.0-20.0)	7 (2.0-33.8)	1.000
**Maximal tumor diameter, cm**				
**Mean** ± **SD**	6.3 ± 2.6	6.6 ± 2.8	6.0 ± 2.6	0.657
**Initial or relapsed, *n* (%)**				1
** Initial**	15 (78.9)	7 (77.8)	8 (80.0)	
** Relapsed**	4 (21.1)	2 (22.2)	2 (20.0)	
**Histology, *n* (%)**				0.37
** IFS**	10 (52.6)	6 (66.7)	4 (40)	
**NTRK-rearranged spindle-cell tumors**	9 (47.4)	3 (33.3)	6 (60)	
**Site, *n* (%)**				0.147
** Legs**	10 (52.6)	5 (55.6)	5 (50.0)	
** Arms**	2 (10.5)	1 (11.1)	1 (10.0)	
** Waist**	1 (5.3)	0 (0.0)	1 (10.0)	
** Hand**	2 (10.5)	2 (22.2)	0 (0.0)	
** Head**	1 (5.3)	0 (0.0)	1 (10.0)	
** Abdominal cavity**	1 (5.3)	1 (11.1)	0 (0.0)	
** Axilla**	2 (10.5)	0 (0.0)	2 (20.0)	
**Distance_metastasis, *n* (%)**				0.211
** Locally advanced**	17 (89.5)	7 (77.8)	10 (100.0)	
** Metastatic**	2 (10.5)	2 (22.2)	0 (0.0)	
**Margins, *n* (%)**				0.777
** R0**	14 (73.7)	6 (66.7)	8 (80.0)	
** R1**	2 (10.5)	1 (11.1)	1 (10.0)	
** R2**	3 (15.8)	2 (22.2)	1 (10.0)	
**Days_to_remission** ± **SD**	63.2 ± 37.5	84.5 ± 37.0	50.4 ± 33.1	0.077
**Long_before_surgery** ± **SD, cm**	2.9 ± 2.7	4.6 ± 3.2	1.4 ± 0.9	0.007
**Remission_befor_surgery, *n* (%)**				0.087
** Yes**	16 (84.2)	6 (66.7)	10 (100.0)	
** No**	3 (15.8)	3 (33.3)	0 (0.0)	
**Teratogenic or disability, *n* (%)**				0.087
** Yes**	3 (15.8)	3 (33.3)	0 (0.0)	
** No**	16 (84.2)	6 (66.7)	10 (100.0)	
** pCR, *n* (%)**				0.087
** Yes**	4 (21.1)	0 (0)	4 (40)	
** No**	15 (78.9)	9 (100)	6 (60)	

### Targeted therapy significantly extended the time-to-treatment failure and EFS

**Figure 1.**
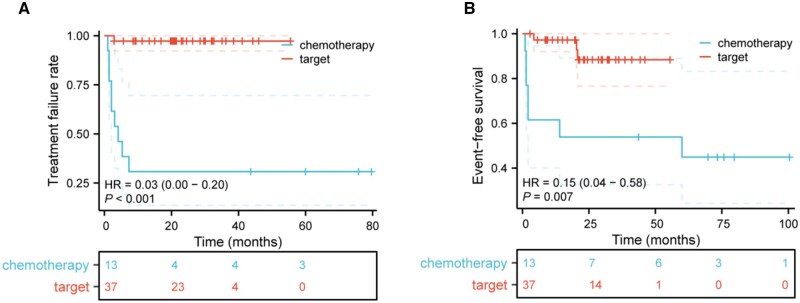
TRK inhibitor therapy prolonged time to treatment failure and event-free survival (EFS). (A and B) Kaplan–Meier analysis of time to treatment failure and EFS in patients receiving chemotherapy (blue) versus TRK inhibitors (red), respectively. *P* < .05 was considered statistically significant.

Targeted therapy markedly reduced the hazard of treatment failure (HR = 0.03, 95% CI: 0.00-0.20, *P* < .001) (Figure 1A). The median time to treatment failure was 4.1 months in the chemotherapy group and was not reached in the targeted group. In addition, overall, 3 EFS events occurred among 37 patients in the targeted group (2 relapses and 1 progression), whereas 8 EFS events occurred in the chemotherapy group (5 progressions and 3 relapses). TRK inhibitors lowered the risk of any EFS event by 85% relative to conventional chemotherapy (HR = 0.15, 95% CI: 0.04-0.58, *P* < 0.007) (Figure 1B).

## Discussion

NTRK fusion-positive sarcomas remain in need of improved therapies given the limited efficacy and unfavorable safety profile of chemotherapy. Using a real-world design with contemporaneous chemotherapy and TRK-inhibitor cohorts, we show that front-line TRK inhibitors exposure is associated with consistently superior clinical outcomes across efficacy, surgical feasibility and early survival metrics.

Fifty patients included in this study had advanced diseases and received first systemic therapy. The treatment failure rate (including disease progression and mutilating surgery) was significantly lower in the TRK inhibitor group (2.7%) than in the chemotherapy group (61.5%, *P* < 0.001). The ORR was also markedly higher in the former (91.9% vs. 53.8%, *P* = 0.006), with 24.3% (9/37) achieving CR. This aligns with findings from the EPI-VITRAKVI study, which reported a failure rate of 19% in chemotherapy-treated historical controls compared with 4% in the Larotrectinib group.^9^ While prior European studies (EpSSG, CWS) reported chemotherapy ORRs of 60%-75%,^5^^,^^6^ approximately 10%-15% of patients still required mutilating surgery. Collectively, these findings underscore the superior efficacy of TRK inhibitors as neoadjuvant options for pediatric NTRK fusion-positive sarcomas, potentially reducing the need for aggressive surgical interventions.

Another consideration is that a higher proportion of patients in the TRK inhibitor group had relapsed disease at baseline compared with the chemotherapy group (37.8% vs. 23.1%). Relapsed tumors are generally considered more resistant to conventional cytotoxic regimens, which could potentially bias the comparison against TRK inhibitors (and, if anything, in favor of chemotherapy). However, despite this unfavorable baseline characteristic, most relapsed patients demonstrated remarkable radiographic and clinical responses to TRK inhibition, and only one newly diagnosed patient in the targeted therapy group exhibited primary resistance. These findings further highlight the robust activity of TRK inhibitors even in the relapsed or refractory setting and are consistent with their clinical advantage over chemotherapy in pediatric NTRK fusion-positive sarcomas.

In addition to recurrence status, the distribution of histological subtypes differed between treatment groups. Specifically, the TRK inhibitors cohort contained a higher proportion of NTRK-rearranged spindle-cell tumors, a histological subtype generally considered less responsive to conventional chemotherapy. Treatment responses vary considerably across different histological subtypes, which might also have influenced the observed outcomes.

For instance, IFS-a tumor that frequently harbors ETV6-NTRK3 fusions- has shown modest responses to chemotherapy in previous European studies. The VA regimen used by the EpSSG yielded an ORR of 68%^5^, whereas the VAC regimen employed by the CWS group achieved an ORR of 75%.^6^ In this study, all 7 IFS patients in the chemotherapy group achieved remission (ORR 100%), which was not significantly different from that of the targeted therapy group (13/14, 92.9%, *P* = 1.00). This discrepancy may be attributable to our choice of a more intensive alkylating agent-containing chemotherapy backbone from the outset. Nevertheless, surgery-related disability and disfigurement, as well as the toxicities of chemotherapy, remain pressing issues to be addressed. 57.1% (4/7) of these patients experienced postoperative disability—such as limping—due to tumor encasement of nerves or muscle. In contrast, none of the patients in the targeted therapy group underwent mutilating surgery.

Conversely, NTRK-rearranged spindle-cell tumors, which in our cohort were predominantly associated with NTRK1 fusions with a diverse spectrum of fusion partners, exhibited poor or absent responses to conventional chemotherapy. None of 5 patients in the chemotherapy group achieved an objective response, and 4 experienced disease progression during treatment. In comparison, the ORR in the targeted therapy group for this subtype reached 95% (19/20). These observations underscore the need for histology-aware treatment strategies and reinforce the importance of molecular profiling to optimize therapeutic decisions in pediatric NTRK fusion-positive sarcomas. Although the TRK inhibitor group included a higher proportion of histologic subtypes typically resistant to chemotherapy, its markedly superior response rate suggests that TRK inhibition has robust activity across histologic subtypes.

Given that surgery is a key determinant of postoperative deformity or disability, we sought to exclude the possibility that the higher incidence of such outcomes in the chemotherapy group was merely attributable to a greater proportion of patients undergoing surgery. We therefore compared the rates of postoperative deformity or disability among patients who underwent surgery in both groups. Although the difference did not reach statistical significance, the incidence in the chemotherapy group was as high as 33.3% (3/9), while no such events occurred in the targeted group. Further analysis revealed that targeted therapy shortened the time to remission by ∼5 weeks and reduced preoperative tumor size to approximately one-third of that observed after chemotherapy. Collectively, these findings indicated that TRK inhibitors were associatedwith more rapid and deeper tumor responses, smaller tumors at the time of surgery, and no mutilating surgery in this selected surgical cohort. Notably, 40% (4/10) of patients in the targeted group achieved pCR following surgical resection. This finding raises the hypothesis that, in carefully selected cases, a surgery-sparing strategy could be explored for patients with exceptional responses to TRK inhibitor therapy, ideally in prospective studies with rigorous imaging and follow-up.

Durable disease control underscores the potential of TRK inhibitors for first-line use. Compared with chemotherapy, TRK inhibitors significantly extended time to treatment failure and EFS. These results are consistent with the faster and deeper responses described above and suggest that TRK inhibitor has not only rapid-onset but also durable effects. No deaths occurred among the fifty patients during follow-up period, thus OS could not be assess.

As a non–randomized, retrospective analysis, the study remains susceptible to unmeasured confounding and selection bias despite the use of contemporaneous control cohorts. The modest overall sample (*n* = 50) and, in particular, the small chemotherapy arm (*n* = 13) restrict statistical precision, widen confidence intervals, and preclude robust multivariable adjustment for all potential covariates. Due to the limited sample size, we were unable to further compare the efficacy of different TRK inhibitors. Furthermore, the use of multicenter data is a double-edged sword. Although drawing cases from 3 tertiary pediatric oncology hospitals improves external validity, center-specific differences in imaging schedules, surgical expertise, and supportive care may have influenced outcomes. Central radiologic and pathologic review was not uniformly available, and standardization of response assessments was retrospective rather than protocol driven. Moreover, the median follow-up of ∼22 months in the targeted group is insufficient to capture late relapses, long-term toxicities, or overall survival. In addition, the study lacked systematic molecular characterisation beyond confirming the presence of an NTRK fusion. Consequently, the influence of fusion partner, breakpoint, or co-mutational landscape on response and resistance could not be explored. Beyond pediatric sarcomas, TRK inhibitors have demonstrated remarkable efficacy across a wide spectrum of NTRK fusion–driven cancers, highlighting their tumor-agnostic potential. Future research should aim to delineate how specific fusion breakpoints and fusion partners modulate sensitivity and resistance to TRK inhibition. Comprehensive molecular characterisation, including detailed mapping of NTRK rearrangements, will be crucial for understanding response heterogeneity and optimising precision-targeted treatment strategies.

Although TRK inhibitors have shown encouraging results, their long-term effects remain to be observed. Clinically, when histopathology indicates a probable diagnosis of IFS rather than rhabdomyosarcoma, early molecular testing and prompt initiation of TRK inhibitor therapy should be strongly considered. This strategy is not only substantially less toxic than conventional chemotherapy but may also serve both diagnostic and therapeutic purposes, as a rapid and profound response to TRK inhibition can help confirm the presence of an underlying NTRK gene fusion while achieving tumor shrinkage and facilitating limb-sparing or function-preserving surgery. Collectively, these findings support the early integration of molecular testing and TRK inhibitor therapy into the management of pediatric NTRK fusion-positive-sarcomas to improve efficacy and preserve function.

## Conclusion

Our real-world cohort demonstrated that TRK inhibitors are highly active in children with NTRK fusion-positive sarcomas. Compared with chemotherapy, TRK inhibitors were associated with markedly fewer treatment failures, a higher ORR, and no mutilating surgeries, thereby enabling function–preserving R0 resections in most patients with localized diseases. These data support TRK inhibitors as a preferred first-line option for this molecularly defined population. Nevertheless, larger prospective, multicenter studies with longer follow-up and integrated molecular profiling are warranted to validate long-term survival benefits and refine subtype-specific treatment algorithms.

## Supplementary Material

oyaf401_Supplementary_Data

## Data Availability

The data underlying this article cannot be shared publicly because they originate from an ongoing clinical trial and represent an incomplete interim dataset. Data will be made available after trial completion, database lock, and primary publication.
